# Skin microbiome & host immunity: applications in regenerative cosmetics & transdermal drug delivery

**DOI:** 10.4155/fsoa-2017-0117

**Published:** 2018-03-28

**Authors:** Kavita Beri

**Affiliations:** 1 Medical Director, Beri Esthetique: Mind Body Skin Integrative Laser Aesthetics Med Spa. Ocean NJ 07712, USA; 2 Adjunct Professor, Department of Biomedical Engineering, Rutgers University, New Brusnwick NJ 08854, USA; 3 Visiting Scientist, Center for Dermal Research, NJ Center for Bio-materials Rutgers NJ 08854, USA

**Keywords:** barrier function, cosmetics, skin, surface microbiome, transdermal drugs

## Abstract

Recent advances in our understanding of the function of the skin and its microbiome have shown that there is a strong symbiotic relationship between the microbiota of the skin and its host immune functions. The dysbiosis or imbalance of the microbiome and other factors that have an influence on the surface microbiota can influence keratinocyte regulation and homeostasis as well as the skin barrier function. In this perspective paper, we review the evidence that connects the skin's microbiome and the barrier function of the epidermis and explore the future potential for applying this unique dialogue in developing innovative cosmetics and transdermal drugs for wellbeing and beauty.

## Lay abstract

The microbiome on the skin has a unique dialogue with the host through the host immune system. This dialogue makes the basis of several host immune responses and help shape the host immunity. In this article, we explore this microbiome and host interaction, and see how this can influence our understanding of skin barrier function, and future applications toward transdermal delivery of topicals.

The primary role of the skin is to serve as a physical barrier, protecting our bodies from potential assault by foreign organisms or toxic substances. The skin is also an interface with the outside environment and, as such, is colonized by a diverse collection of microorganisms – including bacteria, fungi, and viruses [1]. Metagenomic sequencing of the microbiome on many human body sites gives insight into how the microbial biodiversity of the skin is influenced by several factors including environment, ecology, host immunity, genetic predisposition and host lifestyle [2,3]. Skin injury disrupts the homeostasis of the host tissue, and its commensal microbiota. We look at the literature-based evidence that shows a connection in how the dynamic state of the microbiome can influence keratinocyte function and vice versa [2,3]. This perspective aims to explore the influence of the host–microbiome relationship on skin protective functions and barrier stability.

## Invasion of cutaneous pathogen & response of skin ecosystem

Skin microorganisms extend from the surface of the skin to the deeper layers. Almost 25% grow in the deeper layer of the dermis and sebaceous glands [4]. These microorganisms are classified as resident and transient [5]. The resident bacteria transmitted during birth from the mother or acquired from contact with daily life surroundings (animals, plants, persons, chemicals and climates) are long lasting. On the other hand, the exposition to new settings (e.g., changes in the environment due to traveling) leads to the development of new transient microorganisms groups. However, these transient groups are eradicated once back to usual conditions and surroundings. Therefore, each individual has a unique and specific signature of skin microbiota encountered during infancy and stabilized during adulthood [6]. Several papers have documented connections on the microbiome and the host systemic immune system [7].

A tight relationship within this symbiosis regulates pathogen recognition, barrier function, host immune response and evolution of skin diseases like atopic dermatitis, acne, and psoriasis [7,8]. Species of *Staphylococci* are common bacterial colonizers of human skin, and hence, have been studied extensively to see the correlation and influence of their dysbiosis and the impact of this dysbiosis on skin functions. *Staphylococcus epidermidis*, in particular, is the most frequent microorganism isolated from human epithelia and is an essential member of skin resident microflora [9]. *Staphylococcus epidermidis* has an adaptable relationship with its host, and it has the ability to form biofilms that are extremely hard to clear, due in part to the difficulty in bypassing the extracellular matrix, and also to the development of antibiotic resistance and immune resistance [10]. This matrix acts as a physical barrier restricting many antibiotics and chemical diffusion, and as a mechanical barrier restraining immune cell passage.

*Staphylococcus aureus* is another important and prevalent member of the Staph family of resident microbes and is studied extensively due its correlation to multiple cutaneous conditions. It is an excellent model of bacteria, being part of a semiresident flora but able to switch to a pathogen as soon as it is left uncontrolled by other members of resident flora [11]. The specific interaction of this particular microorganism with the host's systemic immune system gives it the ability to attain specific virulence genes easily. *Staphylococcus aureus* produces δ toxins triggering local allergic cutaneous responses which may also prevent wound healing and cause epithelial barrier deterioration [12,13].

More recently, there has been increasing awareness of the importance of fungi and their interactions with the immune system influencing the immune homeostasis and inducing disease. When the chemical composition (pH, pathological sweat secretion) of host epidermis is disrupted, *Malassezia* spp. gains in pathogenicity and releases lipases, phospholipases, and an array of bioactive indoles. These molecules alter the function of the epithelial barrier resulting in immune deregulation and diseases [14].

The ability of the innate immune cells, macrophages, dendritic cells and natural killer cells to communicate with epithelial cells, leading to an effective immune response, is a key feature of the cutaneous immune system [15]. It is of great importance to understand how the cellular and structural composition of the skin dictates the hierarchy of the skin immune response. The epidermis is separated from the dermis by the dermoepidermal junction and from the external environment by the stratum corneum. The latter represents a true barrier of protection. It is composed of cells made up mainly of proteins called corneocytes, whose intercellular space is highly constituted of lipids. The dynamic interaction between all these cells coordinates the immune response. The advances in metagenomic data analysis with the 16s-ribosome compared with regular cultural techniques have helped understand the dysbiosis on the surface of the skin and specifically have been helpful in understanding this dynamic in certain skin conditions such as atopic dermatitis [16]. The skin is a primary immunological barrier to the external environment and has the following interactions. The uppermost ‘corneal layer’ is composed of dead keratinocytes that provide a physical barrier. However, the pathogens can directly access  the interior of the host through skin wounds and by outcompeting the normal flora. Toll-like receptor-bearing cells, keratinocytes and Langerhan's cells recognize pathogens and establish a highly coordinated immune response, which includes: antimicrobial production to neutralize the pathogen; inflammatory mediator secretion to alert the immune cells; activation of innate immune cells such as natural killer cells to induce cell lysis; and/or phagocytosis such as macrophages to engulf pathogens. In the adaptive immune pathway, the immature dendritic cells play a role. The mature dermal dendritic cells migrate into draining lymph nodes to prime T-cell responses to create antigen-specific antibodies through the clonal proliferation of T- and B-cell lymphocytes in the lymph nodes [13]. When the innate immunity and signaling are insufficient to clear a pathogen and to resolve pathogen invasion, the adaptive immune system becomes involved. The coordination between innate and adaptive immunity is assured by dermal dendritic cells, which are professional antigen-presenting cells known as immune system gatekeepers [17].

## Exploring the skin–gut & skin–brain axis through the microbiome's interactions with the host immune system

Defining the deep and intricate connection which the microbiome has with its host leads one to explore the effects of the microbiome on the host's cutaneous and systemic functions – in particular, on those functions influenced by the immune system. A correlation within the skin–gut axis is evident, as shown by connections between psoriasis and Crohn's disease, for example. The crucial interface organs gut and skin have much in common with regards to commensal bacteria. The communication and symbiotic balance of these with microbe-heavy sites is intricate [18].

Gut–skin dysbiosis in many related skin and gut conditions can therefore theoretically be put forward as an explanation for pathophysiology that leads to disruption of barrier functions in the respective organs, so leading to permeability and inflamed states [19]. Much exploration is needed in the field to understand the immunological crosstalk between the skin and gut microbiomes in healthy and diseased states.

The skin–brain axis is also an interesting hypothesis that has been recently examined by connecting post-traumatic stress disorder to skin conditions such as atopy, and exploration is underway to understand how emotional states can affect the skin microbiome and vice versa [20].

Skin cells manufacture and metabolize steroid hormones, peptide neurohormones, and neurotransmitters. Some of these are disseminated further by sweat and sebum [21]. On making contact with cutaneous microbes, they can influence virulence, growth, and adhesion. For example, experimental studies have suggested a relationship between psychological stress-induced increases in local substance P (linked to eczema, acne and barrier dysfunction [22,23]) production [24] and changes in skin microbiota [25,26]. However, some paradigm-shifting studies have provided a different perspective. Here, pathology is not entirely mediated in a unidirectional manner from brain to skin. Recent research has placed epidermal keratinocytes at the forefront of sensory systems, showing that they influence whole-body states and even emotions by generating a variety of hormones and neurotransmitters [27]. This includes the capacity for glucocorticoid production via elements of the local hypothalamic–pituitary–adrenal axis – acting as an independent steroidogenic organ, in addition to the sensors of mechanical stress, temperature and chemical stimuli [28]. Cutaneous cortisol production is stimulated by sin stressors (for example, dryness and barrier disruption); it is possible that this action occurs through activation of inflammatory cytokines such as IL-1β, and has systemic implications [29].

## Conclusion & future perspective: the cosmetic microbiome & regenerative therapies for the skin

We have tapped into a wealth of information on the surface of our skin that can help identify and channel a deeper understanding of skin structure and regulatory functions. This science now serves as a platform for various diverse applications in understanding the host immune system in different diseased states [30]. The future of skin microbiome research will be heavily influenced by a more clear understanding of the resident commensal microbes as a facilitating tool to connect external factors to the host.

A paper published by Nakatsuji *et al*. shows the presence of active bacterial components through 16s-ribosomal and pyrosequencing and in the deeper layers of the skin like the dermis and subcutaneous areas. This raises the possibility of surface microbes interacting with the deeper microbial components, deeper immune cells, and pathways by a complex dialogue. A better understanding of this dialogue will then help us to design cosmetics that can be applied topically but without needing penetration, and that can have deeper effects in the dermal tissue and immune functions of the host skin and body. This study also confirms human skin actively regulates bacterial entry or penetration into dermis through the skin barrier function and antimicrobial peptide (*AMP*) secreted by keratinocytes, and these are perhaps key regulators to maintain dermal microbiome homeostasis [30]. *AMPs* are gene-encoded peptides that comprise a highly conserved component of the innate immune system, and contribute to direct microbial destruction and tissue repair pathways. A study by Plichta *et al*. examined the impact of burn injury on the epidermal barrier and *AMP* production at the donor graft site. They concluded that graft rejection can be as a result of the impairment of the *AMP* regulation and barrier permeability. More studies are needed to clarify the influence of microbes on *AMP* regulation and their impact on improving graft uptake [31]. Certain bacteria in the *Propionibacterium* species are capable of producing their own *AMP*, and this might be of future interest in studying and designing topicals that can influence barrier function and perhaps enhance regenerative pathways through *AMP*.

Another possibility of exploration is looking at the influence of cosmetic products [28,29], such as epidermal exfoliating treatments for anti-aging, and the possibility of designing novel cosmetics that kick-start a regeneration process within the epidermal layer by influencing local immune signals that in turn govern host regenerative pathways. With the evidence of how dysbiosis, or a change in the homeostasis between microbiome and host, can influence host immunity, we can begin the quest to understand the different ways by which this aspect can be further explored so as to fully grasp the regenerative pathways of the skin. Several applications can be cited, including wound healing, and innovative cosmetics that can influence the slowing of cellular aging, as well as influence deeper penetration of actives.

Another avenue in innovation to explore is using drug delivery devices like ultrasound and radiofrequency for delivery of cosmetics [32], and the change in surface microbiome in certain skin conditions like acne. Based on the paper by Nakatsuji [30], we are left with several hypotheses of how altering surface bacteria, with its very complex host relationship, can significantly change host regenerative processes that could also be used for aesthetics and enhancing dermal collagen synthetic activity. The very interesting relationship explored between the skin and gut opens avenues of understanding of the diet, and its influence on the skin.

For centuries, ancient practices like Ayurveda have connected the importance of the gut to overall health, and now, through medical science, we are beginning to notice an interaction between the skin and the gut, and their respective microorganisms, and the connection to a healthy, balanced, immune system. This leads us to speculate that much of ancient eastern science has always seen this unique relationship. Ayurveda also emphasizes the importance of emotional health and its connectivity to the health of the rest of the organs by a dosha evaluation. In the pathways that are modulated in a neuroendocrine-based system as cited in the previous section of the article, we can appreciate how this connection is possible. Vibration science [33] interconnects the ancient philosophy of interconnectivity of all living organisms through energy and it would be fascinating to explore the possibilities of altering the microbiome to influence emotions, especially through cosmetics that can trigger local neuroendocrine pathways or perhaps vibrational healing methods like reiki or sound vibration [27]. Another field of study for understanding the skin–brain axis could be in assessing any impact of meditations and mindfulness in the change of the surface microbiome and looking for changes or improvement in skin conditions or skin aging. Various studies that correlate breathing and yoga to improvement in overall wellbeing can also guide the design of testing this axis more deeply through changes in the microbiome and correlating it to blood antioxidant levels [34,35]. More work is needed in using devices with penetrating active ingredients that affect regenerative and immune pathways that can then stimulate alterations in the deeper layers of the skin microbial components and cause more significant host response.

Finally, we should point to the application of the microbiome and host immune interaction as a possible basis for regulating claims by cosmetic products and perhaps creating guidelines that can make for a safe and clear demarcation of the topical action on the body. Regulatory bodies like the FDA could focus on using the unique host–microbiome interaction post-topical application as a fundamental guideline to regulate cosmetics.

The various possibilities expand our horizons to consider a concept of the ‘cosmetic microbiome’, which can influence the skin–gut–brain relationship [36,37], and therefore result in design of innovative cosmetics and transdermal drugs that change the perception of beauty, health and wellbeing. Cosmetics in the future could have the potential to claim that they can not only make you look good but also that make you ‘feel’ good.

**Executive summary**

- The article provides an overview of the skin microbiome; understanding the specifics and current evidence based on the microbial flora of the skin.
- The microbial interaction with the immune system of the host and establishing influence on the host immunity through innate and adaptive pathways is explained.
- The skin–gut–brain axis is summarized in reference to microbiome–host interaction.
- The future applications in cosmetic innovation transdermal drug delivery and mind body skin approach to transdermal therapeutics for personalization of care is discussed in conclusion of the article.
